# Compatabillty

**Published:** 1860-08

**Authors:** 


### COMPATIBILITY.

BY J. T.

That there are constitutions and temperaments possessing an attraction for each other, and others neutral, and still others between whom there is a repelling power, is a fact that no one having an acquaintance with human nature will for one moment deny.

The conditions are usually recognized at first contact, especially when they are definitely marked. These peculiarities modify all our course of life: by this principle things and persons agreeable are drawn to us, and those disagreeable repelled.

It is true that many persons can so hide or cover up their real character, that in smooth sailing it will not fully appear, and on this account there often seems to be a compatibility, when in reality there is not; in such cases when any thing occurs to develop the true character of each, then the incompatibility appears, and probably with accumulated strength.

If these conditions influence the affairs of every day life, they will certainly operate upon the dentist and his patient. The circumstances are such as to bring out the true character of each, and that oftentimes in a marked degree.

The attractive principle influences the operations of the dentist far more than most persons are aware.

We are familiar with the fact that when we operate for a person for whom we have an aversion, it is in the nature of things impossible to exercise that degree of patience and skill, that can be brought to bear under more favorable circumstances.

If the patient augments the difficulty by fretfulness and irritability, it is impossible for an operator to perform his operations as well as he otherwise could.

The dentist sometimes finds a patient for whom it is a pleasure to work; in such instances he is disposed to prolong the operation sufficiently to accomplish every step perfectly, and do all that is necessary to be done. A feeling pervades the operator under such circumstances that he must perform his work well.

When the dentist knows that his patient will appreciate his operations, and feel grateful to him for his effort and submit kindly to his judgment, and in future will take care of his teeth, and endeavor to obtain the most value from the operations, then it is that the dentist labors under favorable circumstances, and then if ever will he try to make good operations.

But the case is very different if he has a patient for whom he has an aversion, and it may be cross, irritable, illnatured, suspicious, faithless in regard to the efficiency of the work, and will take no care of the teeth in future.

Cross and irritable? Yes, every moment asking if you are not done; accusing you of doing more than is really necessary, just to give them pain; remarking occasionally that they "think dental operations do more harm than good," "and that you are filling a great many more cavities than there are in the teeth;" and that "Dr. B. is a splendid dentist, for he filled my teeth and did not hurt me a particle," when at the same time a plugger will go through Dr. B's. plugs about as readily as through a firmly rolled ball of cotton.

Now, surrounded with such circumstances, there is not an operator living, who can make good operations.

We probably never find a patient in whom is concentrated all the objectionable qualities here enumerated; but occasionally they are found with a considerable number of them, and some repulsive things are sometimes found that are not here enumerated.

For such patients we usually do nothing more than they point out and require, and point out to them just as little as is at all consistent with duty: and that which we do attempt, we endeavor, though through tribulation, to perform as well as possible. It would in some respects be better never to attempt to operate under such circumstances.

